# A gene-editing/complementation strategy for tissue-specific lignin reduction while preserving biomass yield

**DOI:** 10.1186/s13068-021-02026-5

**Published:** 2021-09-03

**Authors:** Hasi Yu, Chang Liu, Richard A. Dixon

**Affiliations:** 1 BioDiscovery Institute and Department of Biological Sciences, University of North Texas, 1155 Union Circle #311428, Denton, TX 76203 USA; 2 Center for Bioenergy Innovation (CBI), Oak Ridge National Laboratory, Oak Ridge, TN 37831 USA

**Keywords:** Saccharification, Lignin modification, Gene editing, CRISPR, Cinnamoyl CoA reductase (CCR1)

## Abstract

**Background:**

Lignification of secondary cell walls is a major factor conferring recalcitrance of lignocellulosic biomass to deconstruction for fuels and chemicals. Genetic modification can reduce lignin content and enhance saccharification efficiency, but usually at the cost of moderate-to-severe growth penalties. We have developed a method, using a single DNA construct that uses CRISPR–Cas9 gene editing to knock-out expression of an endogenous gene of lignin monomer biosynthesis while at the same time expressing a modified version of the gene’s open reading frame that escapes cutting by the Cas9 system and complements the introduced mutation in a tissue-specific manner.

**Results:**

Expressing the complementing open reading frame in vessels allows for the regeneration of Arabidopsis plants with reduced lignin, wild-type biomass yield, and up to fourfold enhancement of cell wall sugar yield per plant. The above phenotypes are seen in both homozygous and bi-allelic heterozygous T1 lines, and are stable over at least four generations.

**Conclusions:**

The method provides a rapid approach for generating reduced lignin trees or crops with one single transformation event, and, paired with a range of tissue-specific promoters, provides a general strategy for optimizing loss-of-function traits that are associated with growth penalties. This method should be applicable to any plant species in which transformation and gene editing are feasible and validated vessel-specific promoters are available.

**Supplementary Information:**

The online version contains supplementary material available at 10.1186/s13068-021-02026-5.

## Background

Lignocellulose biomass is highly abundant, energy-rich, and is constructed of polymers with carbon skeletons suitable for biological funneling into a range of fuels and replacement chemicals to help drive the bio-economy [1–3]. The major component of lignocellulose is secondary cell walls made of cellulose and hemicellulosic polysaccharides, which are impregnated with lignin [4]. Lignins are aromatic heteropolymers, composed mainly of guaiacyl (G) and syringyl (S) units, derived from the monolignols coniferyl alcohol and sinapyl alcohol, respectively [5, 6]. Much smaller amounts of *p*-hydroxyphenyl (H) units derived from *p*-coumaryl alcohol are also often present. More recently, lignins composed exclusively of caffeyl alcohol (C-lignin) have been found in seed coats of some monocot and dicot species [5]. The physical properties of lignin provide strength for plants to stand upright and hydrophobicity to allow efficient water transport through the vascular system [7]. Lignin also acts as a primary, constitutive physical barrier against ingress of pathogens and attack by herbivores [7, 8]. At the same time, the protective chemical and physical properties of lignin present a major impediment to the downstream processing of lignocellulosic biomass for multiple applications [1, 5, 9].

The complexation of lignin to polysaccharides hinders the enzymatic hydrolysis of cell wall polysaccharides into simple sugars during bioprocessing [10, 11]. Because of this, modification of lignin content and/or composition can improve the enzymatic saccharification of biomass [12–22]. However, this is often accompanied by unacceptable reductions in biomass and seed yield [1, 9, 12, 13, 15, 23].

Although the molecular mechanisms underlying lignin modification-induced biomass reductions are still somewhat unclear [23–26], efforts to rescue such defects through recovery of vessel cell integrity have proved successful in the model plant *Arabidopsis thaliana*. To this end, the artificial SECONDARY WALL NAC BINDING ELEMENT of the XYLEM CYSTEINE PROTEASE1 promoter (ProSNBE) has been developed [27]; this confers high expression in both protoxylem and meta-xylem vessels. Tissue-specific complementation of the cinnamoyl CoA reductase 1 (*ccr1*) mutation in the lignin pathway by expression of *ProSNBE:CCR1* resulted in restoration of biomass yield, and a fourfold increase in total sugar yield when compared with wild-type plants [28]. This approach requires transformation of an identified mutant and can therefore be difficult to apply to most bioenergy crops that lack mutant populations. CRISPR/Cas9 technology itself is, however, applicable to biomass crops [29, 30], but generation of lignin pathway mutants by CRISPR/Cas9 and re-transformation to complement in order to restore vessel integrity under a vessel-specific promoter requires removal of the Cas9 protein to avoid cutting of the transformed gene. Theoretically, this could be done by knock-in or replacing with a new vessel-specific promoter to drive the gene encoding the lignin synthesis enzyme. However, the efficiency of CRISPR/Cas9-derived knock-in or replacement is currently very low, and several generations will be required to obtain genetically stable material, thereby limiting the use of this approach [31, 32].

Recent studies have attempted to address the problem of reducing lignin by CRISPR/Cas9-mediated gene editing while maintaining biomass. Approaches include development of a screening method to identify efficient sgRNAs in Arabidopsis which target the enzyme hydroxycinnamoyl CoA: shikimate hydroxycinnamoyl transferase (HCT) coupled with use of an interfascicular fiber-specific promoter, pNST3, to drive CAS9 [33], or using CRISPR/Cas9 to target the *CCR* gene in hybrid poplar by screening for combinations of a null and a haploinsufficient *CCR* allele to obtain a balance between reduced lignin and normal biomass [34].

We here report a simple approach, tested in the model species *A. thaliana*, to promote sugar release by reducing lignin content with maintenance of biomass yield. We generated non-functional CCR1 with the regular CRISPR/Cas9 system, but fused to the vector a ProSNBE promoter driving a version of CCR1 encoding the wild-type protein but with its codons modified to escape cutting of the transgene by the Cas9 system. This method can provide plants with reduced lignin and normal growth in the T1 generation with a single transformation event and no need for complex screening. The phenotype is stable and inheritable.

## Results

### Design of a CRISPR/Cas9 system for lignin reduction in the fiber region

Restoring the lignin content to the xylem vessels in lignin biosynthesis mutants is a proven strategy to maintain lignin reduction in fibers while preserving biomass yield [28, 35–37]. However, as outlined above, current implementations are time consuming and with limited application for many crops and trees.

**Fig. 1:**
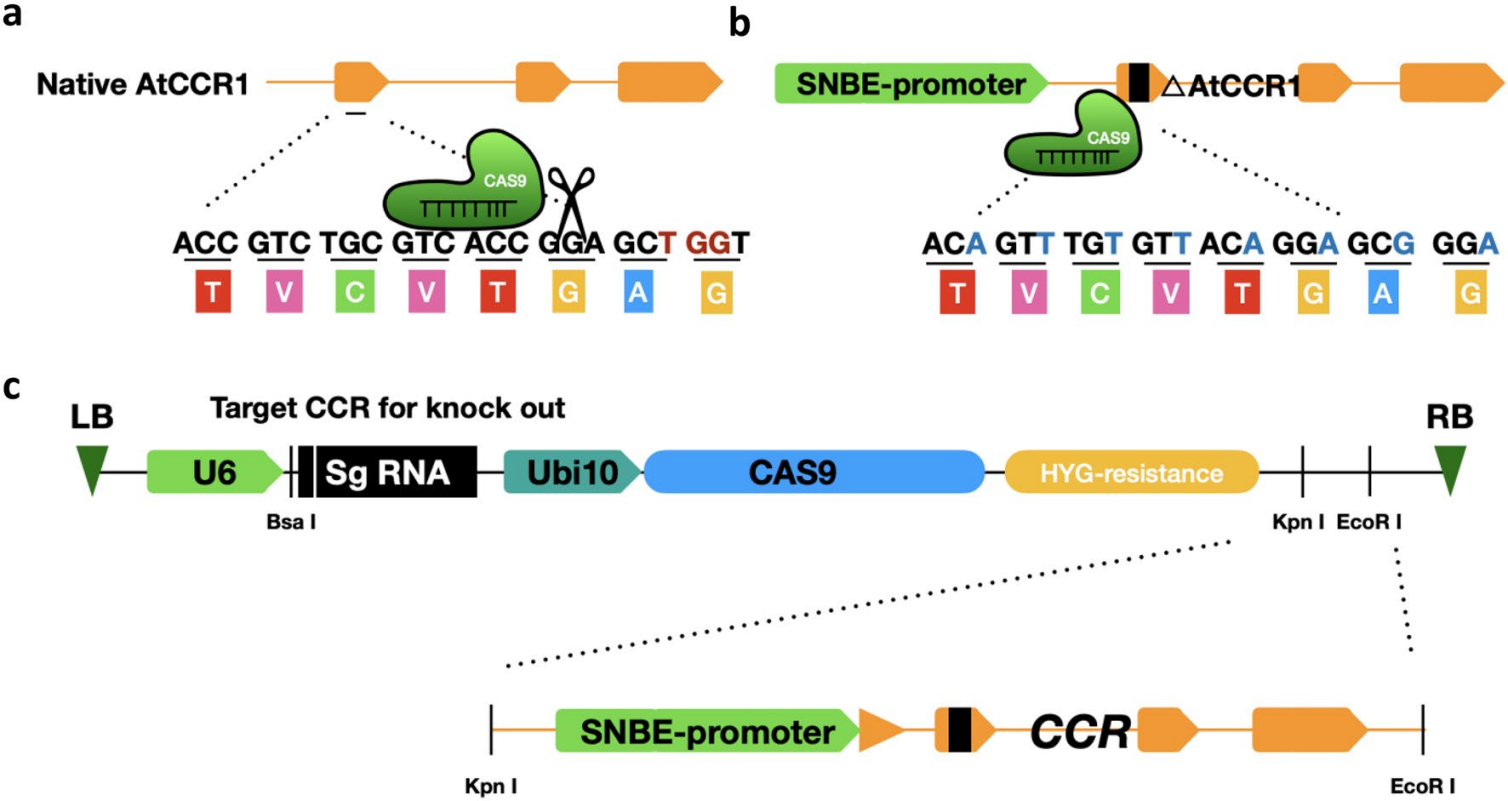
Illustration of the strategy for reducing lignin content by targeting the *AtCCR1* locus using a sgRNA/Cas9 design while restoring CCR1 function to vascular tissues. **a** Diagram of CRISPR/Cas9 system with a sgRNA that targets Cas9 to the native *CCR1* gene. **b** Construct for vessel-specific expression of codon-modified CCR1 that encodes the same protein as native CCR1, driven by the xylem vessel-specific promoter SNBE. **c** Final construct for inserting the ProSNBE: artificial *CCR1* into the pCambia 1300-CAS9 vector with the sgRNA designed for targeting the native *CCR1*

We have a developed a method to generate lignin pathway knock-out mutants and restore the expression of the targeted gene in xylem vessels using one simple construct. The strategy combines targeting Cas9 to the wild-type gene and restoring expression to xylem vessels by co-expressing a version of the targeted open reading frame with altered codon usage to escape recognition by the sgRNA and thereby avoid cutting by the Cas9 system. Figure 1a shows the sgRNA designed to target the *A. thaliana CCR1* gene at the sequence ACC GTC TGC GTC ACC GGA GCT in the first exon, encoding the amino acid sequence T V C V T G A. The sequence for vessel-specific complementation driven by the SNBE promoter encodes the same amino acid sequence with the nucleotides changed to ACA GTT TGT GTT ACA GGA GCG (Fig. 1b). High-frequency off-target mutagenesis induced by CRISPR/Cas9 has been reported in human and mouse cells, but seldom with mismatches over 3 base pairs [38–40]. Furthermore, deep sequencing of a total of 178 off-target sites showed that multiplex targeting in *A. thaliana* is highly specific to on-target sites with no detectable off-target events [41]. In the present case, we introduced six mismatches into the *CCR1*-coding region, and tested 24 T1 transgenic plants to check for off-target editing in the artificial *CCR1*.

### Efficient creation of a null mutation via targeted deletion at the *AtCCR1* locus

Using the scheme in Fig. 1, we designed a sgRNA which can target the *CCR1* gene, and introduced this sgRNA into *pCambia1300-CRISPR/Cas9* to knock out *CCR1.* Next, we cloned the *CCR1*-coding sequence into a T-vector harboring the SNBE promoter, and used a point-mutation kit to change the sequence to avoid targeting by the sgRNA as outlined above. Finally, we inserted *ProSNBE:ΔAtCCR1* into the *pCambia1300-CRISPR/Cas9* vector with endonucleases *Kpn1* and *EcoR1* (Fig. 1c).

**Fig. 2:**
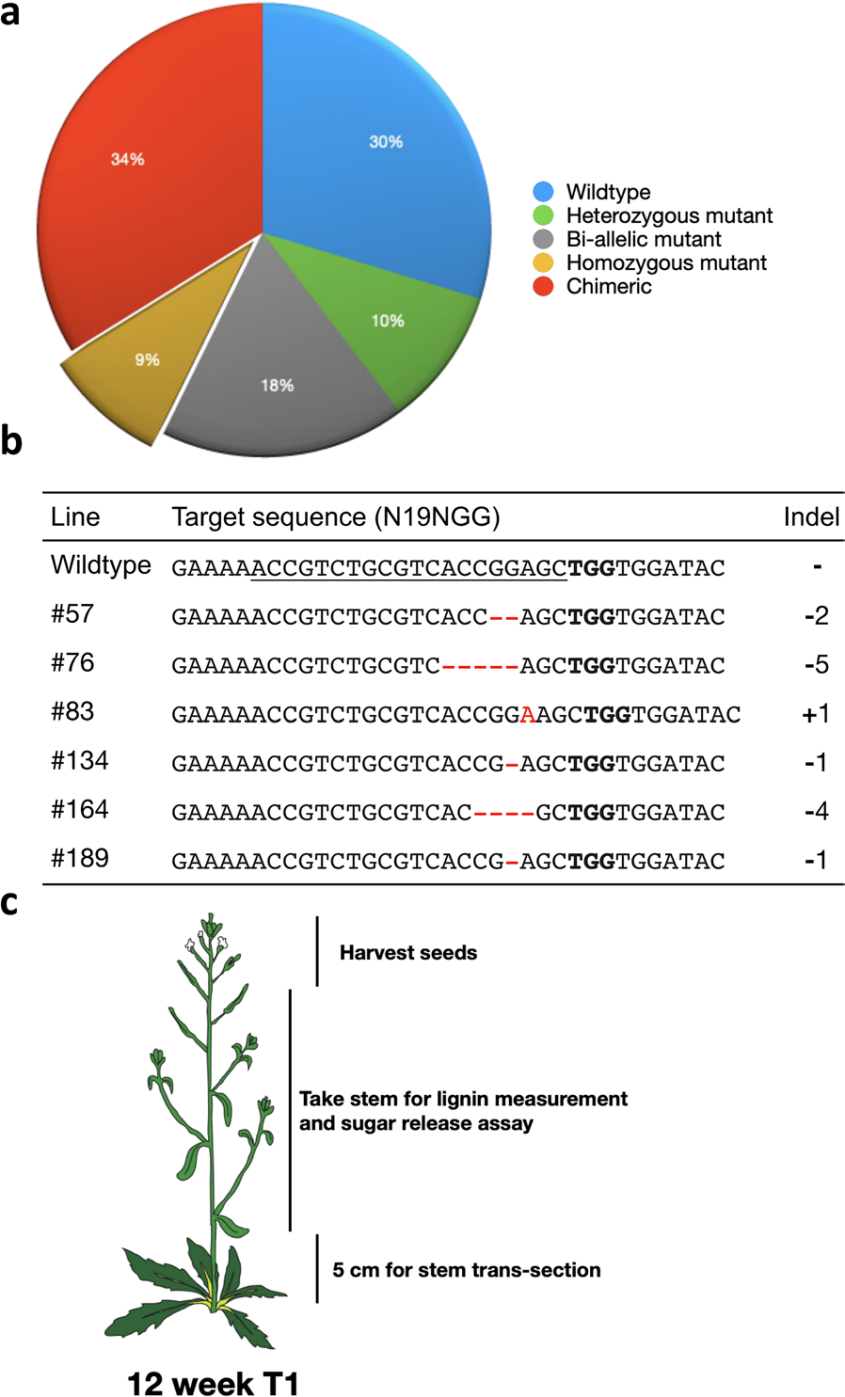
Genotyping of T1 transgenic plants and the distribution of zygosity. **a** Zygosity of T1‐edited plants. Following isolation of leaf DNA, genomic sequences spanning the sgRNA target site were PCR amplified and analyzed by Sanger sequencing. Transgenic plants of each line were classified based on mutation rate at the projected target site. **b** Target sequences of wild-type Col-0 and representative homozygous *ccr1* mutants in Cas9 transgenic lines. Their detection frequencies and the indel patterns (red color) are shown, and the sgRNA (underlined) and protospacer adjacent motif (PAM; bold text) sequences are highlighted. **c** Analysis scheme for gene-edited lines. For 12-week-old T1 homozygous and bi-allele *ccr1* mutants, seeds were harvested from individual seedlings, the bottom 5 cm of inflorescence stems were taken for imaging of cross-sections for lignin deposition, and the main regions of inflorescence stems were harvested for lignin measurement and sugar release assay

One hundred and ninety-two independent T1 plants transformed with *pCambia1300-CRISPR/Cas9-ProSNBE:ΔAtCCR1* constructs were genotyped by Sanger sequencing of the target region. The sequencing results were analyzed by Tracking of Indels by Decomposition (TIDE), a tool which identifies the types and frequency of mutations in the region around the projected editing site [42]. The genotypes of the T1 plants are summarized in Fig. 2a. We found that 57/192 T1 plants (~ 30%) were wild type, with no mutation detected in the endogenous *CCR1* gene; 65/192 (~ 34%) were chimeric, namely wild type combined with more than two kinds of mutation in the target region; 19/192 (~ 10%) were heterozygous mutants, namely wild type combined with 1 kind of mutation in the target region; 34/192 (~ 18%) had no wild-type peak but two (bi-allelic) or more than two (tri-allelic) mutations; and 17/192 T1 plants (~ 9%) were homozygous mutants.

### Growth phenotype of T1 plants with homozygous *CCR1* mutation

We randomly selected six seedlings (genotypes shown in Fig. 2b) from the 17 homozygous mutants for further analysis; these represented one single nucleotide insertion and five deletions of 1, 2, 4 or 5 nucleotides. All these mutations changed the reading frame of CCR1, and disrupted gene function by creating premature termination codons at the start of the CCR1 N-terminus (Additional file 1: Fig. S1). We took the basal 5 cm of the stems of 12-week T1 plants for examination of cross-sections to visualize lignin deposition, and the remainder of the main stem for lignin measurement and sugar release assay after harvesting the T1 seeds (Fig. 2c).

**Fig. 3:**
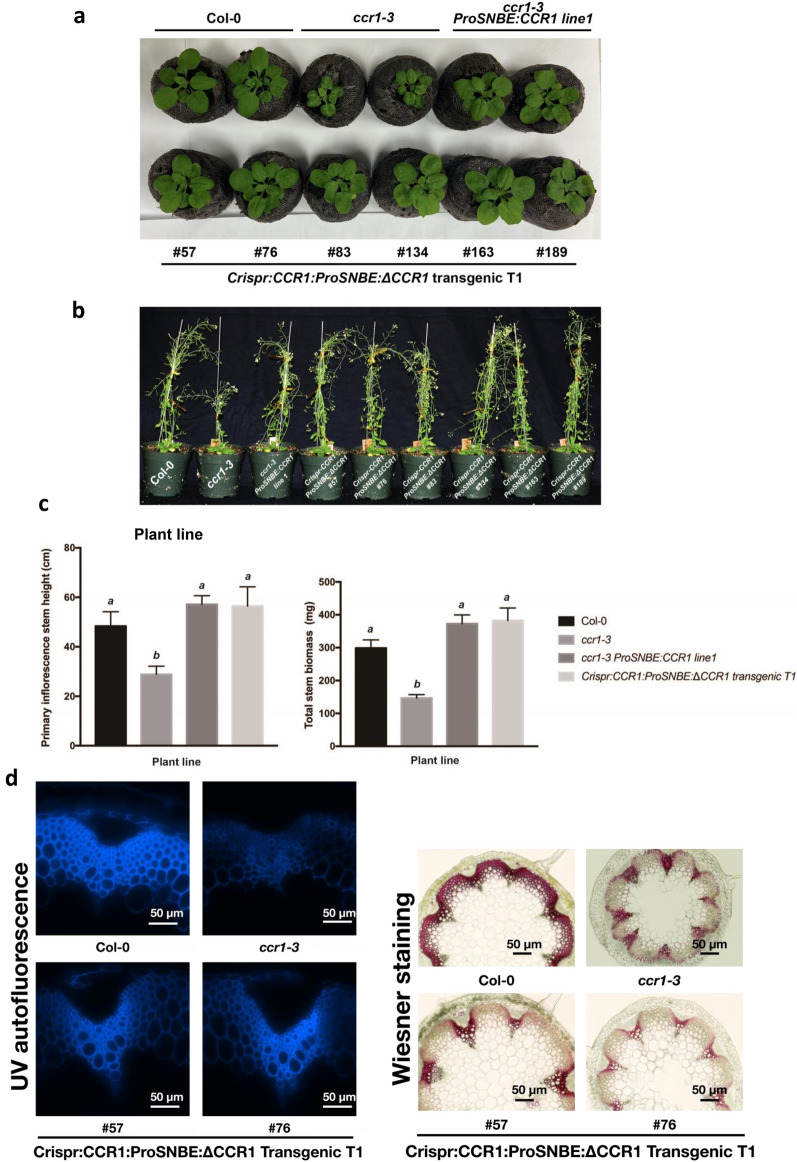
Phenotyping of T1 transgenic plants. **a** Phenotypes of 4 weeks Col-0, *ccr1*–3, *ccr1–3* ProSNBE:CCR1 and transgenic Crispr:CCR1:ProSNBE:ΔCCR1 T1 lines. **b** Phenotypes of 12-week-old plants of the above lines. **c** Primary inflorescence stem height and biomass measurements of 12-week-old plants of the above lines. **d** Lignin deposition in inflorescence stems of the above lines. Transverse stem sections are shown with lignin autofluorescence and Wiesner staining. See Additional file 1: Fig. S4 for photographs of additional T1 plants. Bars = 200 µm. In **c**, bars represent means and standard deviations, *n* = 6. Letters above bars indicate statistical significance by one-way ANOVA, *p* < 0.05

Two-week-old T1 seedlings with homozygous *Crispr*-mediated *CCR1* mutation with co-expression of *ΔCCR1* did not show the distinctive *ccr1* phenotype of small rosettes and shrunken leaves (Fig. 3a); the growth phenotype was the same as wild type. An sgRNA targeting the first exon of CCR1 should generate a strong allele, so we selected *ccr1–3,* a strong allele [9], as an additional control, and transformed it with *ProSNBE:CCR1* as a check for the vessel-specific complemented phenotype. The growth characteristics of the *ccr1–3/ProSNBE:CCR1* and *Crispr:CCR1:ProSNBE:ΔCCR1* transgenic lines appeared identical (Fig. 3C, D). Furthermore, T2-generation homozygous *Crispr:CCR1:ProSNBE:ΔCCR1* edited lines that had lost the *Crispr:CCR1:ProSNBE:ΔCCR1* construct showed a strong Crispr-generated *ccr1*-negative growth phenotype, and growth rescue relied on the presence of *Crispr:CCR1:ProSNBE:ΔCCR1* (Additional file 1: Fig. S2)*.*

For further analysis, we transferred the T1 seedlings to larger pots, grew the plants for another 8 weeks (Fig. 3b), then measured the primary inflorescence stem height and the total weight of the Col-0, *ccr1–3, ccr1–3 ProSNBE:CCR1* and *Crispr:CCR1:ProSNBE:ΔCCR1 T1* lines. The height of the primary inflorescence stem of *Crispr:CCR1:ProSNBE:ΔCCR1* T1 lines was equal to that of the wild type and *ccr1–3 ProSNBE:CCR1* lines, nearly twofold higher than that of the *ccr1–3* T-DNA insertion mutant (Fig. 3b, c). Likewise, the total stem weight of *ccr1 ProSNBE:CCR1* and *Crispr:CCR1:ProSNBE:ΔCCR1* T1 lines was equal to that of the wild type, approximately twofold higher than that of the *ccr1* T-DNA insertion mutants (Fig. 3c). Homozygous lines of Crispr:CCR1:ProSNBE:ΔCCR1 exhibited a lodging phenotype, but some individuals among the bi-allele or weak mutants in CCR1 showed less lodging (but also less lignin reduction, see below).

### *Crispr:CCR1:ProSNBE:ΔCCR1* T1 lines show reduced lignin levels and improved saccharification efficiency

**Fig. 4:**
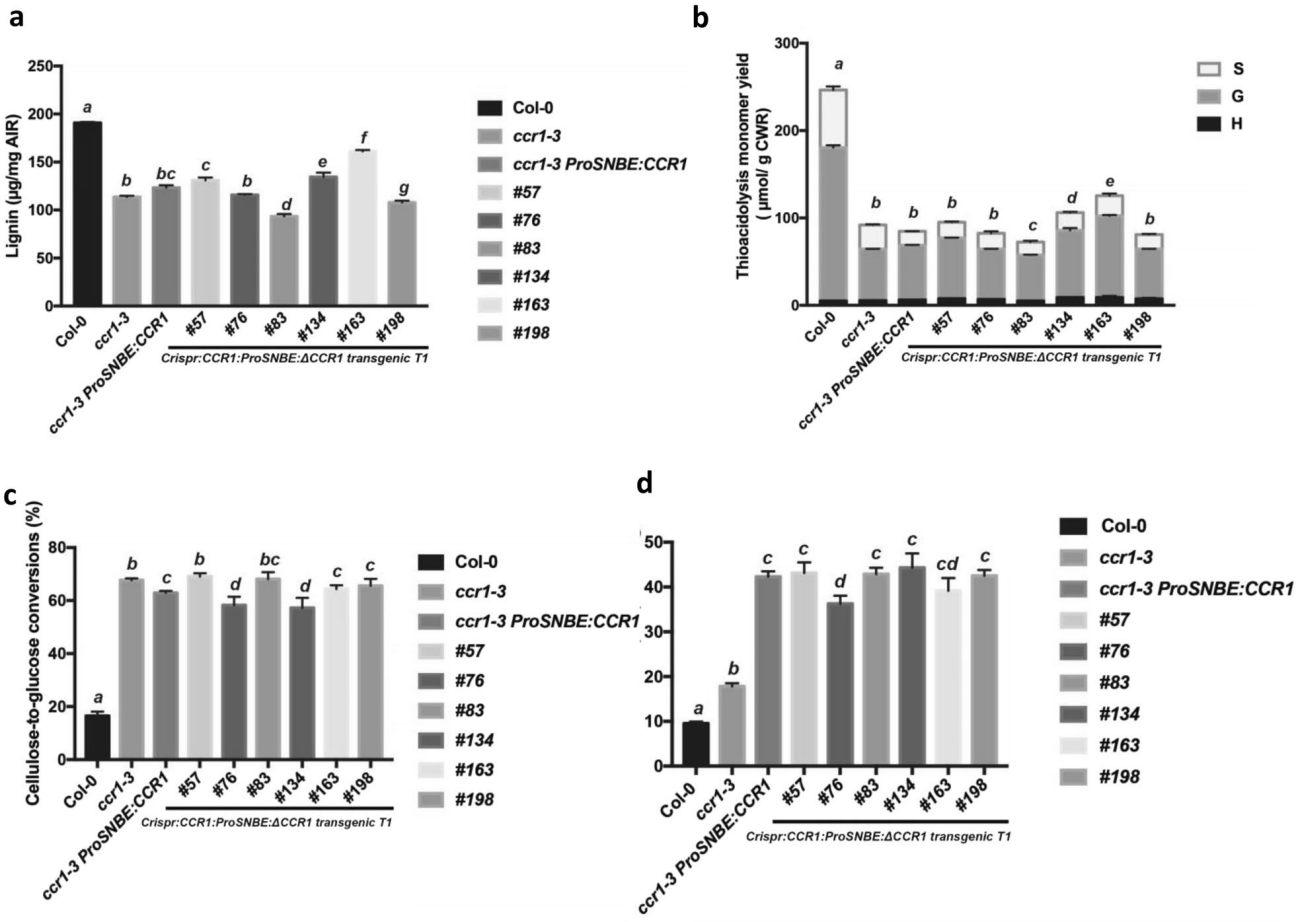
Biochemical phenotypes of T1 *ccr1* homozygous knock-out plants. **a** Total lignin levels of Col-0, *ccr1–3, ccr1–3* ProSNBE:CCR1 and transgenic Crispr:CCR1:ProSNBE: ΔCCR1 T1 lines as determined by the acetyl bromide method. **b** Lignin monomer composition of the above lines as determined by thioacidolysis and GC/MS after TMS derivatization. **c** Saccharification efficiencies of the above lines. Cellulose-to-glucose conversions were determined after 10 days of saccharification of senesced inflorescence stems. **d** Glucose release after 10 days of saccharification per total stem biomass. Samples were saccharified without pretreatment. Bars represent means and standard deviations of four biological replicates. Letters above bars indicate statistical significance by one-way ANOVA, *p* < 0.05

The xylem tissue in wild-type *A. thaliana* plants contains large, open vessels, and the interfascicular fibers are massively lignified (Fig. 2d). In contrast, the xylem tissues in *ccr1–3* mutants show an overall reduction in lignin deposition, as observed by both UV autofluorescence and Wiesner (phloroglucinol) staining, and develop irregularly shaped and collapsed vessels [12, 43, 44]. The xylem vessels of *Crispr:CCR1:ProSNBE:ΔCCR1* T1 lines showed an intense coloration on Wiesner staining and contained large open vessels similar to those of the wild type and the *ccr1–3 ProSNBE:CCR1* lines [28] (Fig. 4d). The xylary fibers also appeared to be lignified. However, the interfascicular fibers showed reduced lignin deposition similar to the *ccr1–3* mutant line.

To examine the lignin content of stems of *Crispr:CCR1:ProSNBE:ΔCCR1 T1* lines, we utilized the acetyl bromide spectrophotometric method [45, 46] for estimation of total lignin and the thioacidolysis method [47, 48] to reveal lignin monomer composition. The strong reduction in total lignin level observed in the *ccr1–3* mutant was partially rescued in the *ccr1–3 ProSNBE:CCR1* line and in some, but not all, of the *Crispr:CCR1:ProSNBE:ΔCCR1* T1 lines (Fig. 4a). However, total thioacidolysis yields (H + G + S) were strongly reduced in all lines compared to wild type (Fig. 4b). Larger effects on thioacidolysis yield compared to acetyl bromide lignin level are commonly seen in lignin genetic modification studies [28, 33, 49]. The two methods measure different features of the bulk lignin, with thioacidolysis only releasing β-*O*-4-linked monomer units [50, 51]. Our data are consistent with the lack of a significant increase in bulk lignin on vessel complementation of the *A. thaliana ccr1–6* mutant allele as previously reported [28].

H lignin monomers constituted less than 2% of the total thioacidolysis-released monomer unit in wild-type plants (Additional file 1: Fig. S3). H lignin content increased in *ccr1–3*, *ccr1–3* ProSNBE:CCR1, and *Crispr:CCR1:ProSNBE:ΔCCR1* T1. Furthermore, the S/G ratio decreased in the *ccr1–3* mutant compared to the wild type, and this decrease was maintained in the *Crispr:CCR1:ProSNBE:ΔCCR1* T1 plant as well as the *ccr1–3 ProSNBE:CCR1* lines (Additional file 1: Fig. S3).

We next compared the saccharification efficiency without pretreatment for wild-type, *ccr1–3*, *ccr1–3 ProSNBE:CCR1* and *Crispr:CCR1:ProSNBE:ΔCCR1* T1 lines, using the upper portions of the main stems. The cellulose-to-glucose conversion for *Crispr:CCR1:ProSNBE:ΔCCR1* T1 was approximately threefold higher than that of wild-type stems, similar to that of the *ccr1–3* mutant and the *ccr1–3* ProSNBE:CCR1 lines (Fig. 4c). The beneficial effect of the growth restoration arising from vessel-specific complementation can be seen in the total yields of sugar release per plant, which were over fourfold compared to only approximately twofold in the *ccr1–3* mutants (Fig. 4d).

### Lignin reduction and improved saccharification efficiency are heritable in *Crispr:CCR1:ProSNBE:ΔCCR1* lines

Posttranscriptional gene silencing by RNA interference has been used for development of commercial low-lignin germplasm [52]. However, RNAi is not always heritable in plants [53]. Furthermore, RNAi of plant transgenes can sometimes occur without a trigger, as a stochastic phenomenon that shows limited epigenetic inheritance [54]. Therefore, there are advantages to avoiding RNAi approaches for fiber-specific targeting of lignin for biomass improvement.

Using a fiber‐specific promoter to drive CAS9 expression with a sgRNA efficiently targeted the *A. thaliana HCT* gene to reduce lignin content in fibers [33]. However, the T1 plants were chimeras, and it was not determined whether the favorable phenotype was stable and inheritable. Our approach generates homozygous mutations in the T1 generation, and the xylem vessels rescue relies on a linked transgene, suggesting that lines homozygous for the *Crispr:CCR1:ProSNBE:ΔCCR1* locus should provide stable lignin reduction without biomass loss.

**Fig. 5:**
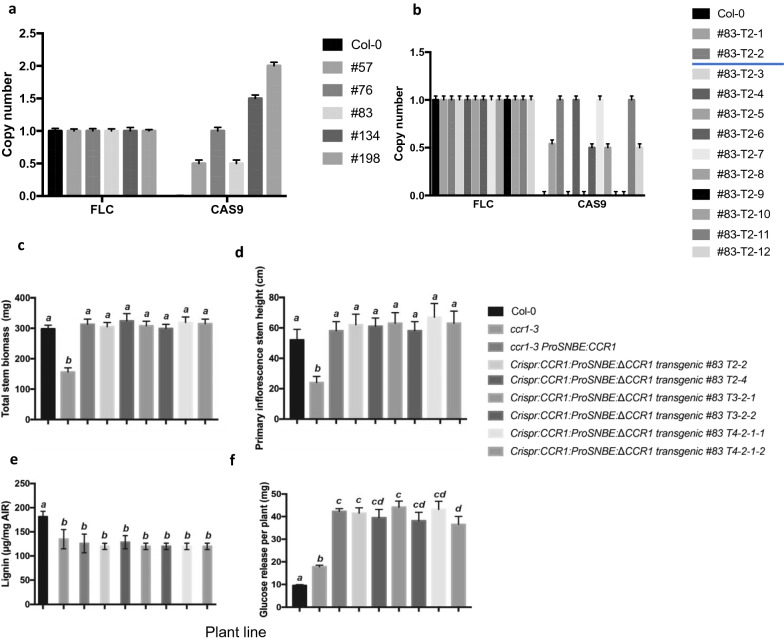
The phenotype of lignin reduction with biomass recovery is stable in the T2, T3 and T4 generations. **a** Cas9 copy number tested by qPCR for the transgenic T1 lines; the #57 and #83 lines are single copy insertion lines. **b** Selecting the single homozygous lines (underlined blue) in the T2 generation from #83 T1 seeds. **c** The total stem biomass of Col-0, *ccr1–3, ccr1–3 ProSNBE:CCR*1 and transgenic *Crispr:CCR1:ProSNBE: ΔCCR1* T2, T3 and T4 lines. **d** The primary inflorescence stem height of the above lines. **e** The lignin content of the above lines. **f** The glucose release per seedling (based on stem inflorescence) of the above lines. In **c**–**f**, bars represent means and standard deviations, *n* = 6. Letters above bars indicate statistical significance by one-way ANOVA, *p* < 0.05

To test this, we first identified single insertion lines among *Crispr:CCR1:ProSNBE:ΔCCR1* T1 plants, using qPCR of the *Cas9* sequence to determine transgene copy number [55]. Using the *FLC* gene as an endogenous homozygous single gene control, we found that lines #57 and #83 possessed single heterozygous transgene insertions (Fig. 5a). To select homozygous *Crispr:CCR1:ProSNBE:ΔCCR1* lines, seed from T1 generation #57 and #83 T1 seeds were collected and sown into soil. The T2 progeny showed segregation of wild-type and *ccr1*-like phenotypes. After genotyping, we found that all seedlings with the *ccr1–3* phenotype had lost the *Crispr:CCR1:ProSNBE:ΔCCR1* transgene. qPCR analysis confirmed that *Crispr:CCR1:ProSNBE:ΔCCR1* lines 83-T2-2, 83-T2-4, 83-T2-7, and 83-T2-11 were homozygous (Fig. 5b). After 12 weeks growth their seeds were collected, stem length and weight measured, and stem material stored at − 80 °C for further analysis. Seeds of line 83-T2-2 were continued to T4, and material collected after 12 weeks as before. The stem height and weight of the *Crispr:CCR1:ProSNBE:ΔCCR1* lines was stable through the T4 generation (Fig. 5c, d). Likewise, the decrease in acetyl bromide lignin content was stable through four generations at around the same level as observed in the *ccr1–3* mutant (Fig. 5e), with a corresponding stable increase in saccharification measured as cell wall glucose equivalents released per plant (Fig. 5f).

### Bi-allelic T1 lines also show lignin reduction and high glucose yield without biomass loss

**Fig. 6:**
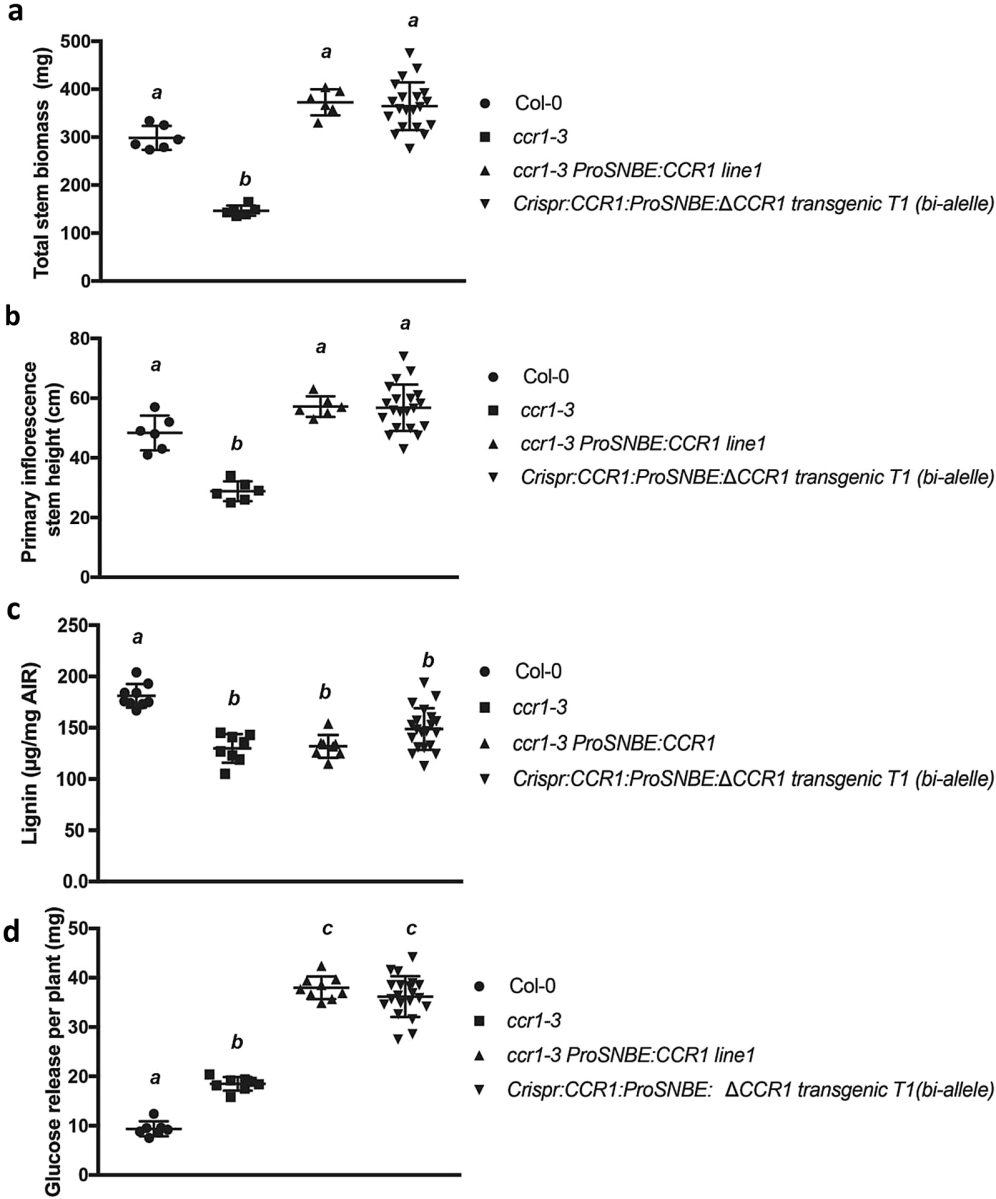
The lignin reduction and biomass recovery phenotype can be detected in T1 bi-allele plants. **a** The total stem biomass of Col-0, *ccr1–3, ccr1–3 ProSNBE:CCR*1 and transgenic *Crispr:CCR1:ProSNBE: ΔCCR1* bi-allele lines. **b** The primary inflorescence stem height of the above lines. **c** The lignin content of the above lines. **d** The glucose release per seedling of the above lines. Data points show individual plants. Bars represent means and standard errors

Eighteen % of the *Crispr:CCR1:ProSNBE:ΔCCR1* T1 lines we generated were bi-allelic, meaning that although the lines may not be homozygous, the wild-type version of *CCR1* is not present, suggesting that such lines may behave similarly to homozygous *Crispr:CCR1:ProSNBE:ΔCCR1* transgenic lines. To test this hypothesis, we measured the primary stem height, total stem weight, lignin content, and glucose yield per plant. The stem weight and size of bi-allelic T1 plants was the same as wild type (Fig. 6a, b), the average lignin level the same as in the *ccr1–3* mutant (Fig. 6c), and, most importantly, the average glucose release was about four times higher than wild type and two times higher than in *ccr1–3* (Fig. 6d).

## Discussion

In hybrid poplar, CCR down-regulation has been shown to yield up to 161% improvement in ethanol yield per unit of biomass [56]. However, the strong CCR RNAi down-regulated poplar lines exhibit large biomass reductions. Suppressing lignin biosynthesis specifically in the fiber region can maintain growth while providing more sugar release in poplar [57]. The present method would provide a quick approach to test the best transgenes for improving saccharification efficiency in a tree or crop species assuming that vessel complementation could overcome the negative growth phenotype. Both CRISPR-gene editing and a suitable vessel-specific promoter are available in poplar, for example [29, 57]. The method could also be used more generally in model, and potentially crop species, to determine, in cases where constitutive gene knock-down or knock-out results in yield penalties, which tissue requires wild-type expression of the targeted gene; in this case, the codon-modified transgene would have to be tested with a range of tissue-specific promoters known to be functional in the chosen species.

Theoretically, the same results might be obtained by direct use of a fiber-specific promoter to drive an antisense or hairpin sequence that targets the lignin biosynthesis gene. However, this may not provide a stable phenotype, especially after several generations, which is a drawback of the antisense and RNAi system [53].

Model species such as Arabidopsis and Medicago with mutant collections that cover most of the genome would not require the CRISPR component of the present strategy, and tissue-specific complementation in such cases is already established [28]. However, for most crops and trees such mutant resources are not available, and complementing existing gene-edited lines would require removal of the Cas9 and a second round of transformation to provide the vessel-specific expression with an experimentally validated promoter. The method described here should prove time-saving for basic studies in crops. Its limitation for commercial application, however, is that the Cas9 and the complementing vessel-specific transcript are on the same construct which, although simplifying development of homozygous progeny lines, means that is not possible to segregate out the Cas9 in subsequent generations. Furthermore, strong lignin down-regulation in fibers can lead to lodging, as in the present case, and it will therefore be necessary to balance improved processing ability with agronomic performance in selecting lines for further trait development.

## Conclusions

In conclusion, we have developed a simple method to obtain, in one generation after a single transformation event, plants with reduced lignin level and higher cell wall glucose release per plant, with no accompanying reduction in biomass yield. In our experiments with *A. thaliana*, 27% of T1 plants showed this advanced phenotype, we could quickly get homozygous transgenic plants with simple genotyping, and the phenotype was stable and inheritable. Further experiments will be necessary to show the utility of this approach in other diploid species, and whether it can be readily translated to forage or bioenergy crops such as alfalfa (*Medicago sativa*) and switchgrass *(Panicum virgatum*) with more complex ploidy levels. As presently formulated, the method is advantageous for basic studies, but approaches that allow for removal of the Cas9 may be necessary to facilitate deregulation of transgenic lines for commercial application.

## Methods

### Plant growth and transformation

*Arabidopsis thaliana* plants were germinated on 1/2 Murashige and Skoog (MS) agar medium with sucrose (15 g/L) at 4 °C for 3 days and transferred to a growth chamber at 22 °C with a photoperiod of 16 h light/8 h dark for 2 weeks. Seedlings were then transferred to soil and growth continued under the same conditions. Transformation of *A. thaliana* was performed by the floral dip method [58]. Transgenic plants were isolated on 1/2 MS medium containing 50 μg/mL hygromycin, and positive seedlings transfer to soil “cookies”. Positive T1 transgenic plants were used for further analysis.

### Gene cloning and plasmid construction

The guide RNA (sgRNA) was designed with Geneious R7 to target the first exon of CCR1, and cloned into pCambia1 300-35S-Cas9 after the AtU6 promoter [59]. The CCR1 open reading frame was PCR amplified from Col-0 cDNA and cloned into pMD20 vector (Takara). The artificial SNBE promoter contained three copies of XCP1-SNBE1 fused to the CaMV 35S minimal promoter (from 246 to 21), as described previously [60]. To construct this synthetic promoter, a 103-bp construct containing the restriction sites for *XbaI* and *NdeI* was first synthesized as a long primer by Invitrogen (Life Technologies). After dilution to 20 nM, this was mixed with 5 µL each of forward and reverse primers (Additional file 1: Table S1), followed by annealing with PCR, down from 95 to 25 °C at 0.1 °C per second. The artificial SNBE promoter was cloned into the *XbaI-* and *NdeI-*digested pMD20 T-vector containing the AtCCR1 coding sequence using T4 ligase. Mutations at the sgRNA target site of AtCCR1 were generated using pMD20 containing *ProSNBE-AtCCR* with a Site-Directed Mutagenesis Kit (NEB, Cat. # E0554S), to make the artificial *CCR1* with wild-type amino acid sequence but altered codons to avoid cutting by the CRISPR system. *ProSNBE:ΔAtCCR1* was PCR amplified and cloned into pCambia1300-35S-Cas9 with the sgRNA targeting native CCR1 using *KpnI* and *EcoI*.

### Genotyping

For Sanger sequencing, DNA was extracted from leaves 3 weeks after planting. Amplification used a forward PCR primer 500 bp upstream of the sgRNA target site, located on the native CCR1 promoter, and a reverse primer 400 bp downstream of the sgRNA target site. The native CCR1 can be isolated by these 2 primers but the transgenic CCR1 cannot. To obtain better sequencing signal, an independent primer 400 bp upstream of the sgRNA target site was used for sequencing. All primers are listed in Additional file 1: Table S1. The sequencing results were uploaded and analyzed for mutation rate with TIDE (https://tide.deskgen.com/) [42], using wild-type samples of CCR1 as the reference. The homozygous and bi-allele mutant results were double checked with Geneious R7.

### Microscopy analyses

The basal internodes of inflorescence stems of 12-week-old plants were collected. Free-hand cross sections of *A. thaliana* inflorescence stems were stained with 0.5% phloroglucinol (w/v) (Sigma-Aldrich) in 12% (v/v) HCl for 3 min, and immediately observed under a bright-field and UV-B microscope.

### Lignin analyses

Mature inflorescence stems from independent 12-week-old *A. thaliana* Col-0, *ccr1* mutant and transgenic plants were harvested after collecting seeds and ground in liquid nitrogen to a fine powder to prepare the alcohol-insoluble residue (AIR) as previously described [46]. After de-starching [61], the crystalline cellulose content and monosaccharide composition were analyzed according to a previously published protocol [62]. The lignin content was determined as described [46]. Lignin composition was determined by an optimized thioacidolysis method [63]. Approximately 2 mg of dry stem material was used for analysis per replicate, and four biological replicates were analyzed.

### Saccharification assays

Inflorescence stems were collected after harvesting seeds, chopped into 2–4 mm pieces, 10-mg stem samples placed into 2-mL Eppendorf tube with steel beads, and the tissue ground at low temperature. Cell wall residue (CWR) was obtained by heating the crushed samples in water at 98 °C, moving the residues into 2-mL glass vials, extracting sequentially with ethanol, chloroform, and acetone, and drying the remaining CWR under vacuum. The amount of cellulose in the CWR was estimated colorimetrically [9]. Saccharification assays (without pretreatment) were performed as described previously, with a 10-day incubation period and enzyme added every 2 days. To estimate the amount of glucose released, the glucose oxidase (GOD)–peroxidase (POD) method was used [64].

## Supplementary Information

**Additional file 1: Figure S1.** Recovered mutations in native CCR1 loci. **Figure S2.** The growth rescue relies on the presence of *Crispr:CCR1:ProSNBE: ΔCCR1.* **Figure S3.** Lignin composition in wild-type, *ccr1–3*, *ccr1–3* ProSNBE:CCR1 and *Crispr:CCR1:ProSNBE:ΔCCR1* T1 plants. **Figure S4.** Lignin deposition patterns in inflorescence stems of T1 transgenic plants. **Table S1.** Primers used in the present work.

## Data Availability

The data supporting the conclusions of this article are included within the article and its additional files.
