# Supplementary material for: A gene-editing/complementation strategy for tissue-specific lignin reduction while preserving biomass yield
Source: Biotechnol Biofuels. 2021 Sep 3;14:175. doi: 10.1186/s13068-021-02026-5 (PMC8417962; doi:10.1186/s13068-021-02026-5)
Supplement: Supplementary file 1 — Additional file 1: Figure S1. Recovered mutations in native CCR1 loci. Figure S2. The growth rescue relies on the presence of Crispr:CCR1:ProSNBE: ΔCCR1. Figure S3. Lignin composition in wild-type, ccr1–3, ccr1–3 ProSNBE:CCR1 and Crispr:CCR1:ProSNBE:ΔCCR1 T1 plants. Figure S4. Lignin deposition patterns in inflorescence stems of T1 transgenic plants. Table S1. Primers used in the present work. [file 13068_2021_2026_MOESM1_ESM.docx]

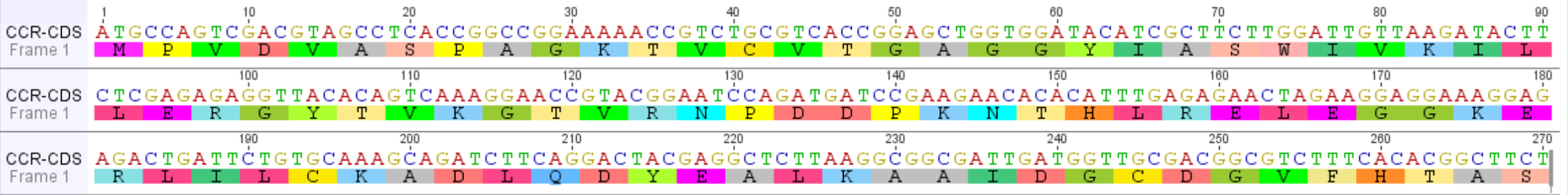

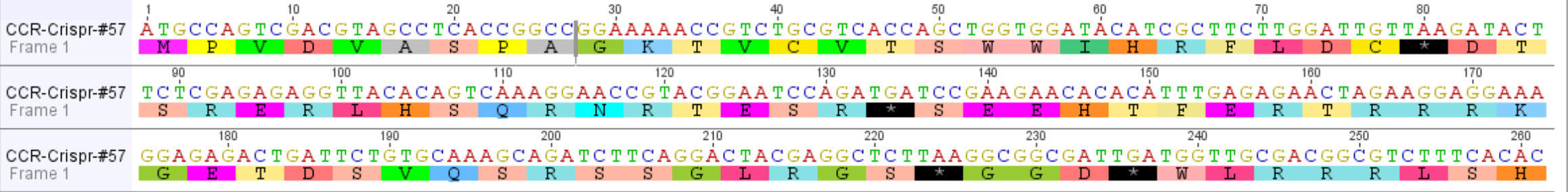

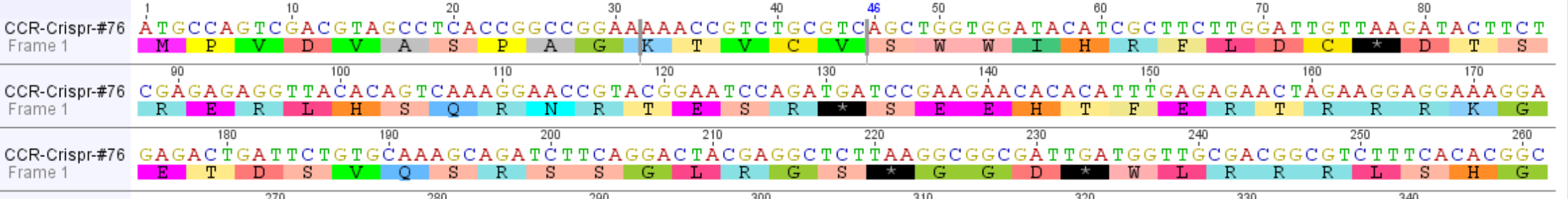

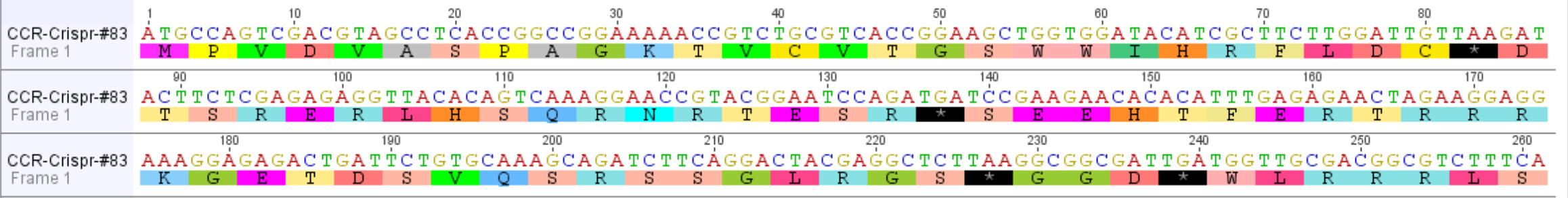

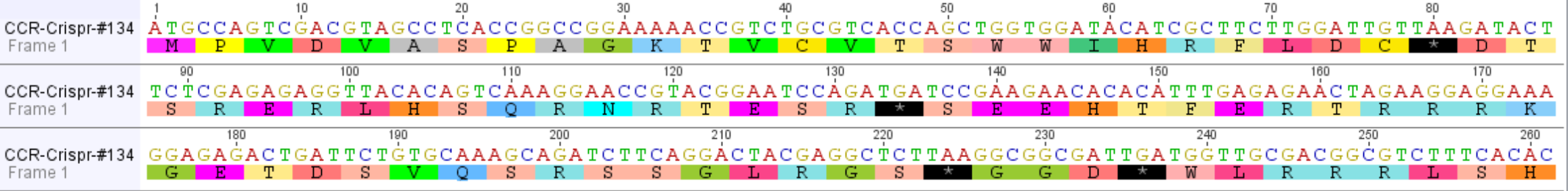

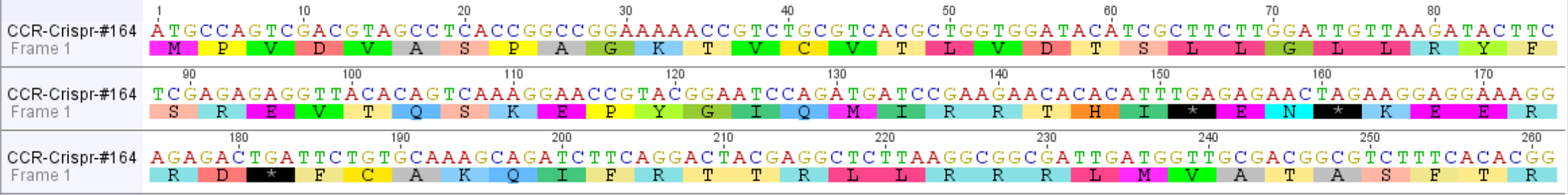

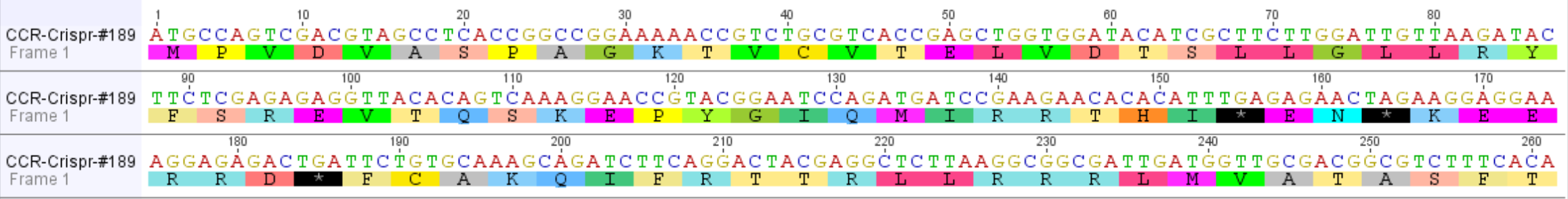


**Figure S1. Recovered mutations in native *CCR1* loci.** All the selected mutants change the reading frame of the edited *CCR1* target, and disrupt gene function by creating premature termination codons (PTCs) at the beginning of the *CCR1* N- terminal region. The black boxes with star show stop codons. The wild-type sequence is shown at the top of the panel.


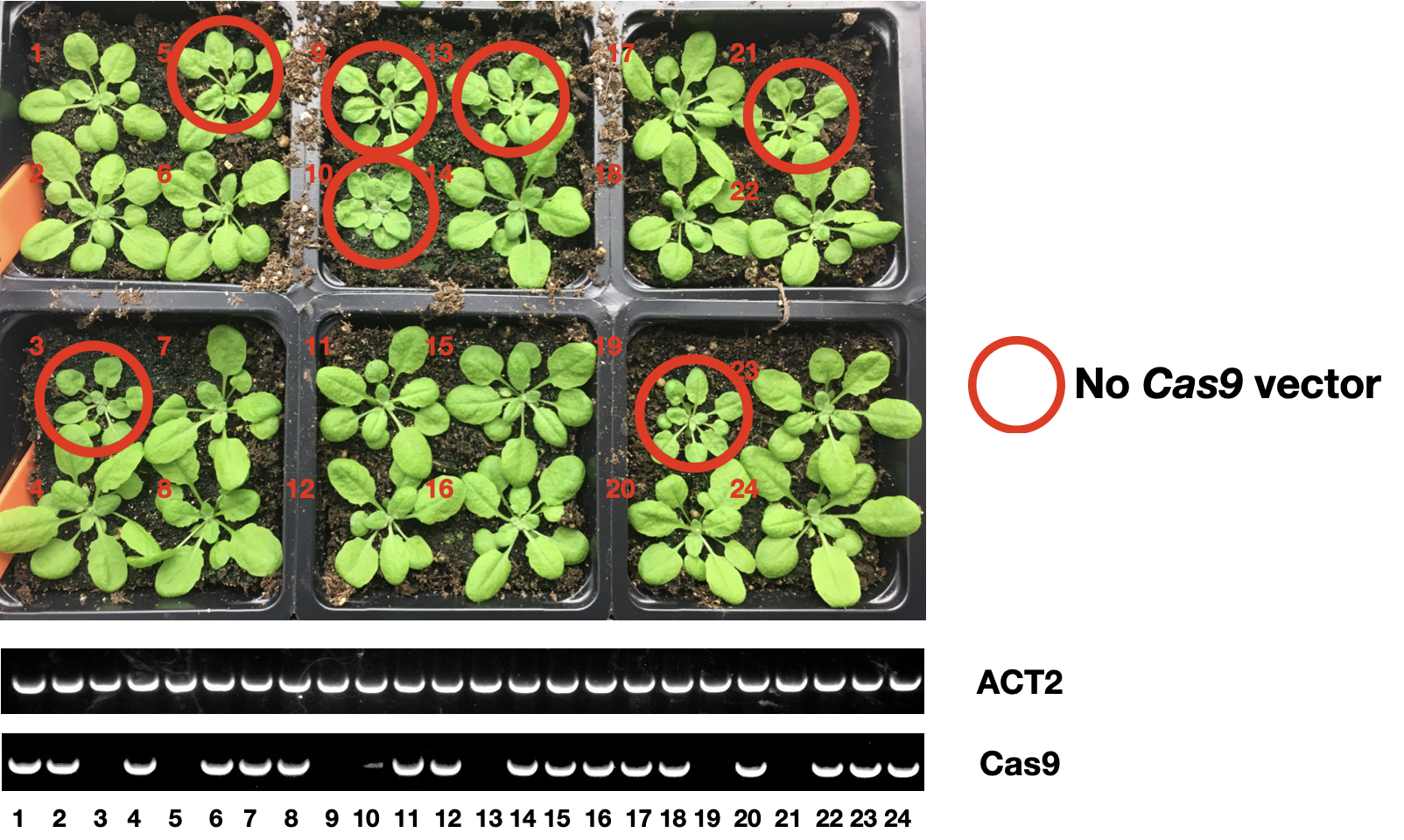


**Figure S2. The growth rescue relies on the presence of *Crispr:CCR1:ProSNBE: ΔCCR1.*** Photographs of T2 seedlings from a cross between *Crispr:CCR1:ProSNBE: ΔCCR1 #8,* which contains a single transgene insertion, and the homozygous *ccr1* mutant used for the segregation test. The seedlings without the transgene construct show the typical leaf shrinking and small size phenotype characteristic of *ccr1-3*.


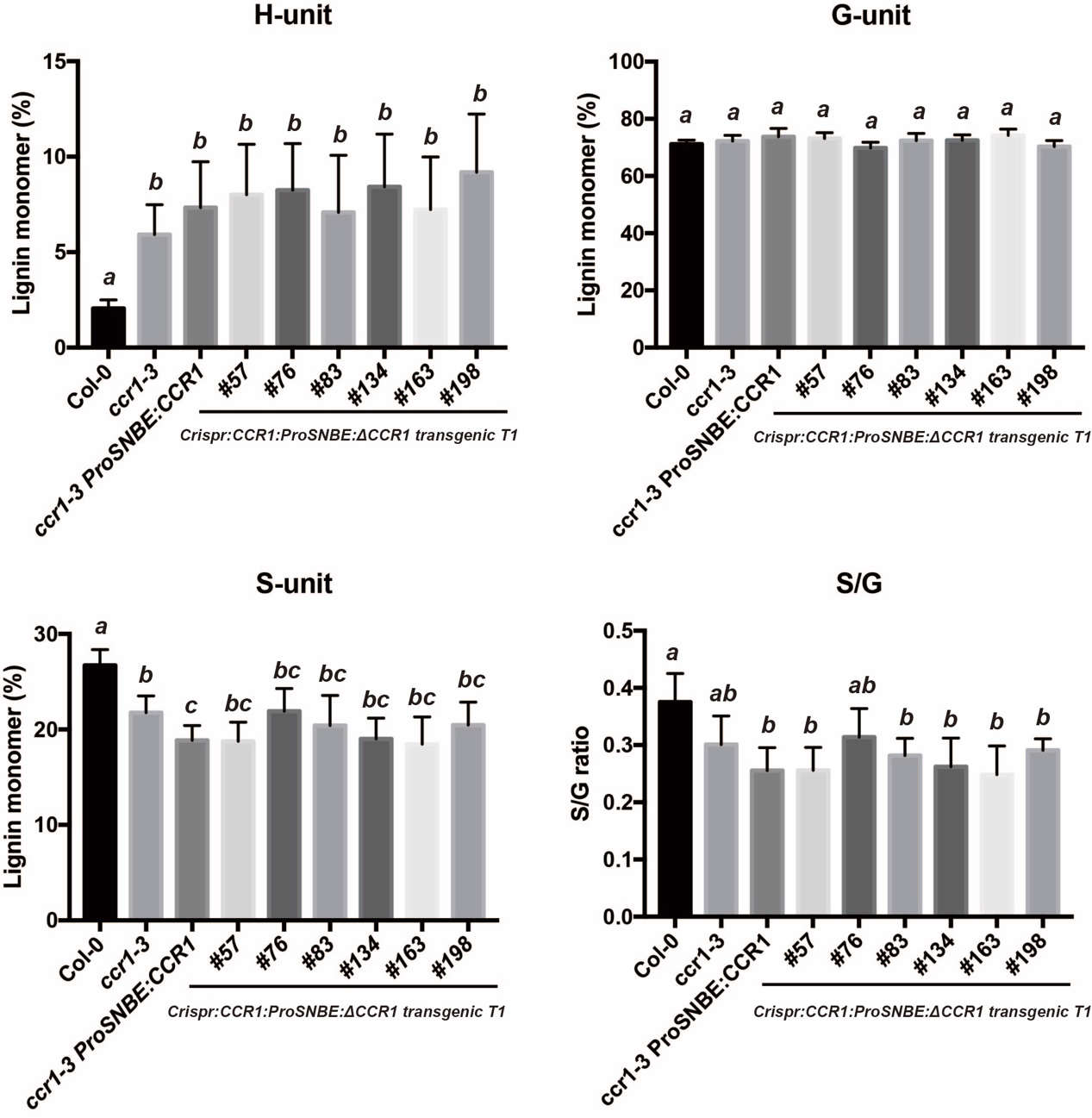


**Figure S3. Lignin composition in wild-type, *ccr1-3*, *ccr1-3* ProSNBE:CCR1 and *Crispr:CCR1:ProSNBE:ΔCCR1* T1 plants.** The lignin composition was determined by thioacidolysis. The relative proportions of the different lignin monomer units were calculated based on the total thioacidolysis yield (including the minor nonconventional lignin units). S/G ratio was calculated based on the absolute values for S and G. Bars represent means and standard deviations of 4 biological replicates. Letters above bars indicate statistical significance by one-way ANOVA, p <0.05.


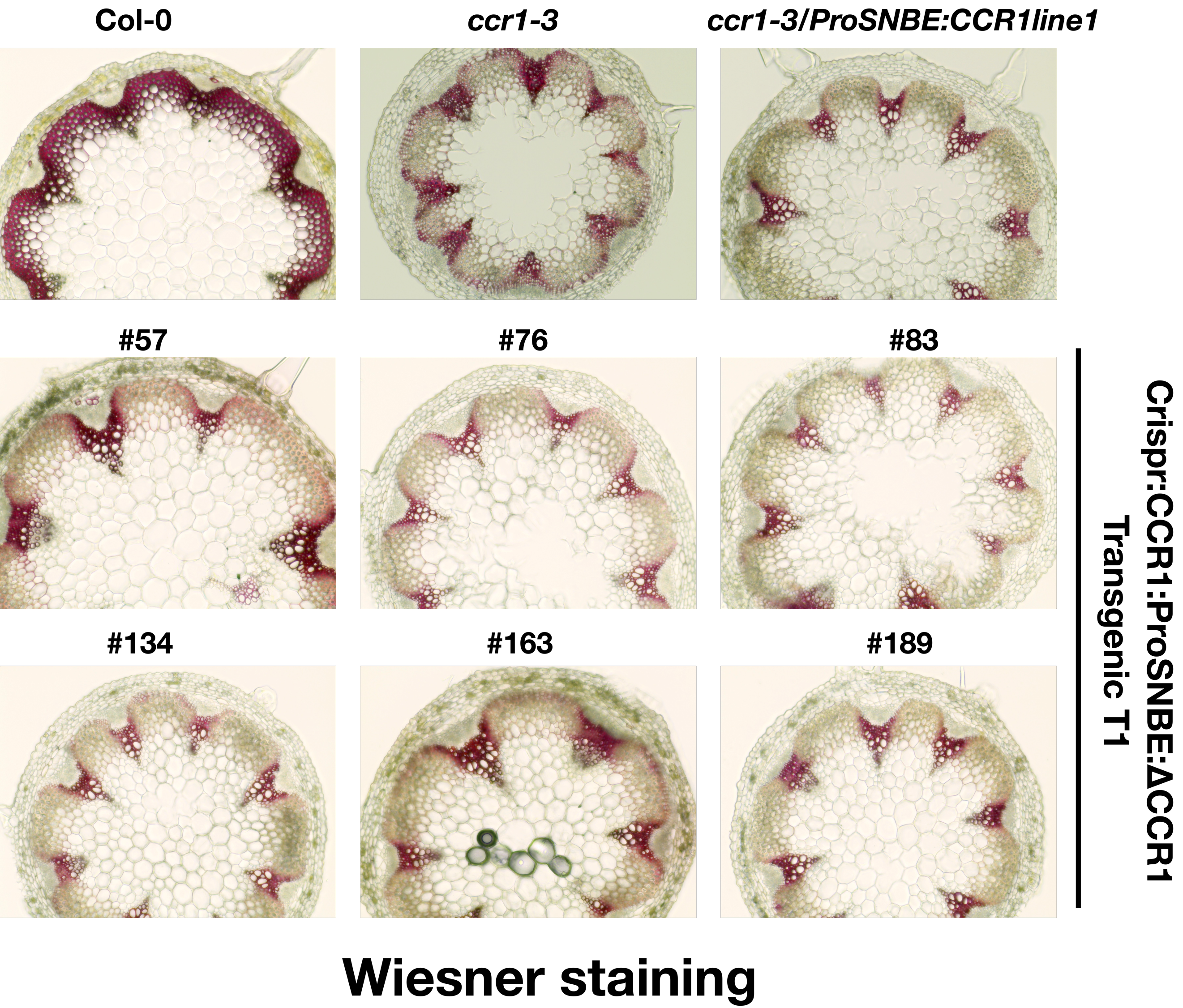


**Figure S4. Lignin deposition in inflorescence stems of T1 transgenic plants.** Transverse stem sections are shown with lignin autofluorescence (above) and Wiesner staining (below). Bars = 200 µm.

**Table S1. Primers used in the present work**
